# Engineered receptors for soluble cellular communication and disease sensing

**DOI:** 10.1038/s41586-024-08366-0

**Published:** 2024-11-14

**Authors:** Dan I. Piraner, Mohamad H. Abedi, Maria J. Duran Gonzalez, Adam Chazin-Gray, Annie Lin, Iowis Zhu, Pavithran T. Ravindran, Thomas Schlichthaerle, Buwei Huang, Tyler H. Bearchild, David Lee, Sarah Wyman, Young-wook Jun, David Baker, Kole T. Roybal

**Affiliations:** 1 Department of Microbiology and Immunology, University of California San Francisco, San Francisco, CA USA; 2 Department of Biochemistry, University of Washington, Seattle, WA USA; 3 Joint Graduate Program in Bioengineering, University of California San Francisco and University of California Berkeley, San Francisco, CA USA; 4 Department of Otolaryngology, University of California San Francisco, San Francisco, CA USA; 5 Department of Pharmaceutical Chemistry, University of California San Francisco, San Francisco, CA USA; 6 Helen Diller Family Comprehensive Cancer Center, University of California San Francisco, San Francisco, CA USA; 7 Institute for Protein Design, University of Washington, Seattle, WA USA; 8 Graduate Program in Biological Physics, Structure and Design, University of Washington, Seattle, WA USA; 9 Howard Hughes Medical Institute, University of Washington, Seattle, WA USA; 10 Parker Institute for Cancer Immunotherapy, San Francisco, CA USA; 11 Chan Zuckerberg Biohub, San Francisco, CA USA; 12 Gladstone UCSF Institute for Genetic Immunology, San Francisco, CA USA; 13 UCSF Cell Design Institute, San Francisco, CA USA; 14 Present Address: Penn Medical Scientist Training Program, University of Pennsylvania, Philadelphia, PA USA

**Keywords:** Applied immunology, Protein design

## Abstract

Despite recent advances in mammalian synthetic biology, there remains a lack of modular synthetic receptors that can robustly respond to soluble ligands and, in turn, activate bespoke cellular functions. Such receptors would have extensive clinical potential to regulate the activity of engineered therapeutic cells, but so far only receptors against cell-surface targets have approached clinical translation^1^. To address this gap, here we adapt a receptor architecture called the synthetic intramembrane proteolysis receptor (SNIPR) for activation by soluble ligands. Our SNIPR platform can be activated by both natural and synthetic soluble factors, with notably low baseline activity and high fold activation, through an endocytic, pH-dependent cleavage mechanism. We demonstrate the therapeutic capabilities of the receptor platform by localizing the activity of chimeric antigen receptor (CAR) T cells to solid tumours in which soluble disease-associated factors are expressed, bypassing the major hurdle of on-target off-tumour toxicity in bystander organs. We further apply the SNIPR platform to engineer fully synthetic signalling networks between cells orthogonal to natural signalling pathways, expanding the scope of synthetic biology. Our design framework enables cellular communication and environmental interactions, extending the capabilities of synthetic cellular networking in clinical and research contexts.

## Abstract (web summary)

A synthetic receptor platform that enables mammalian cells to respond to soluble factors allows specific signalling networks to be precisely controlled, with a variety of therapeutic applications.

## Main

The fundamental basis of biochemical signal transduction lies in the ability of cells to produce, sense and react to small diffusible molecules. This capability allows them to coordinate intricate functions and respond to environmental stimuli beyond their immediate vicinity. For instance, morphogens have a crucial role in shaping the three-dimensional pattern of an embryo during development^2^, whereas cytokines are involved in shaping cell state changes and recruiting a diverse array of immune cells to the site of disease^3^. The ability to mimic these natural systems and interface with soluble factors in synthetic biological systems would enable engineered cells to integrate signals from distant sources and activate therapeutic programs. Furthermore, artificial signalling molecules could provide privileged communication channels between cells to specifically engage engineered cells after administration to patients. Using these bio-orthogonal channels of communication, engineered cells could promote large-scale cellular coordination, facilitating complex multicellular behaviour and delivering synergistic therapeutic benefits.

Despite this potential, progress towards developing sensitive, robust and modular biosensors for soluble factors has been limited. CAR T cells capable of sensing transforming growth factor-β (TGFβ) and other soluble factors have been demonstrated^4^; however, they can induce only a native T cell transcriptional response and a subset of T cell functions, such as cytokine secretion^5^. Immune-cell activities that drive inflammation and cytotoxicity are not always desirable, especially in scenarios in which the cell is simply a smart delivery agent for therapeutic molecules. In addition, strategies that use endogenous stimulation-responsive promoters to drive payload transgene expression are often hampered by the relatively weak activity of such promoters in primary immune cells^6,7^. The MESA receptor platform couples modular ligand sensing to custom transcriptional output, but achieving high sensitivity and dynamic range in therapeutic cell types remains a challenge, and reliance on viral components and a multi-chain architecture complicate translation^8^. TanGo^9^ and ChaCha^10^ are G-protein-coupled receptor (GPCR)-fused protease architectures that offer an alternative approach but still require multiple components, which can constrain the use of therapeutic delivery vehicles such as lentiviral or adeno-associated viral vectors. Moreover, these GPCR-based designs lack the flexibility in ligand selection that is inherent to receptors using modular binding domains (for example, single-chain fragment variables; scFvs). The PAGER system is more modular than its TanGo predecessor, using an scFv to direct receptor function, but retains a more complex multi-component architecture that includes a viral protease for transcription factor release^11^. The OCAR platform also relies on co-delivery of two receptor chains to allow for ligand-induced dimerization and activation^12^. A compact, single-chain receptor capable of modularly sensing soluble factors would overcome the limitations of current systems and unlock the potential for engineered receptors to coordinate therapeutic genetic programs in clinically relevant cell types.

**Fig. 1:**
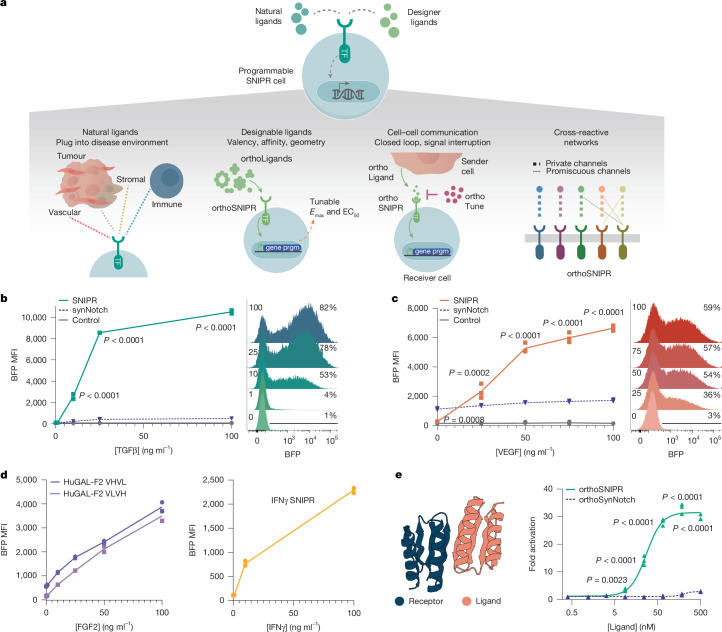
Engineering versatile synthetic receptors capable of sensing natural and engineered soluble ligands. **a**, Clinical and research applications for engineered receptors that are capable of soluble ligand detection. prgm, program; TF, transcription factor. **b**, Left, activation of a TGFβ-responsive SNIPR or synNotch driving a BFP reporter circuit in primary human CD3^+^ T cells after addition of recombinant human TGFβ1 (*n* = 2 technical replicates). Statistics calculated using two-way analysis of variance (ANOVA) with Šídák’s multiple comparisons test; depicted significance corresponds to the comparison of SNIPR versus synNotch. Right, representative histograms of reporter expression level at the indicated concentrations of TGFβ. ****P < 0.0001. MFI, mean fluorescence intensity. **c**, Induction (left) and representative histograms (right) of a VEGF-sensing SNIPR→BFP circuit in primary human T cells through recombinant human VEGF (*n* = 3 technical replicates). The indicated significance corresponds to the comparison of SNIPR versus synNotch; two-way ANOVA with Šídák’s multiple comparisons test. **d**, Receptor-mediated induction of a BFP reporter gene in primary human CD3^+^ T cells using recombinant human FGF2 (left) or IFNγ (right) (*n* = 2 technical replicates). **e**, Left, structural model of the de-novo-designed LHD heterodimer used to establish a bio-orthogonal receptor–ligand pair. Right, fold activation over background of an orthogonal ligand-responsive SNIPR or synNotch driving a BFP reporter circuit in Jurkat T cells through the introduction of orthoLigand C1-active (*n* = 3 technical replicates). Depicted significance corresponds to the comparison of SNIPR versus synNotch; two-way ANOVA with Šídák’s multiple comparisons test. Source Data

Ideally, receptors used for soluble factor detection must operate with high fidelity, generating strong signals in the on state while minimizing basal signalling in the off state. These receptors should also be compact to enable efficient delivery to relevant immune cell types and single-chain to minimize potential issues related to subunit stoichiometry. The synthetic Notch receptor (synNotch) represents a prototypical engineered receptor, using cleavage by endogenous γ-secretase to release its transcription factor after binding to a cell-surface ligand, and showing robust activation in therapeutically relevant cell types including CAR T cells^13,14^. We developed a receptor with a Notch-based architecture, called the synthetic intramembrane proteolysis receptor (SNIPR), which provides a compact and readily tunable scaffold for custom signal transduction along the Notch cleavage paradigm^15^. The ability of SNIPRs to respond to ligand binding robustly and selectively despite the omission of the Notch LNR regulatory domains, which are widely thought to be the key determinants of Notch activation, implies that SNIPRs can use an alternative signalling pathway that bypasses the mechanosensing filter that prevents Notch and synNotch from detecting soluble ligands. We set out to investigate whether the SNIPR system could be adapted to sense soluble ligands, which could enable a wide variety of applications (Fig. 1a).

Our findings confirm that SNIPRs can use an alternative signalling pathway, allowing for robust and selective responses to soluble ligand binding. SNIPRs have the unique capability of interacting with a range of physiological or synthetic ligands, whether they are tethered or soluble. This versatile feature makes them an asset to the current array of cell-engineering tools, with potential applications in cancer therapy, and engineering development. We show that T cells engineered with SNIPR technology can effectively react to soluble factors and drive therapeutic payload production in animal models of solid tumours. Moreover, we show that SNIPRs can serve as fundamental building blocks for constructing modular bio-orthogonal communication systems in cells that can operate independently of natural signalling pathways.

## SNIPRs can detect soluble ligands

**Extended Data Fig. 1:**
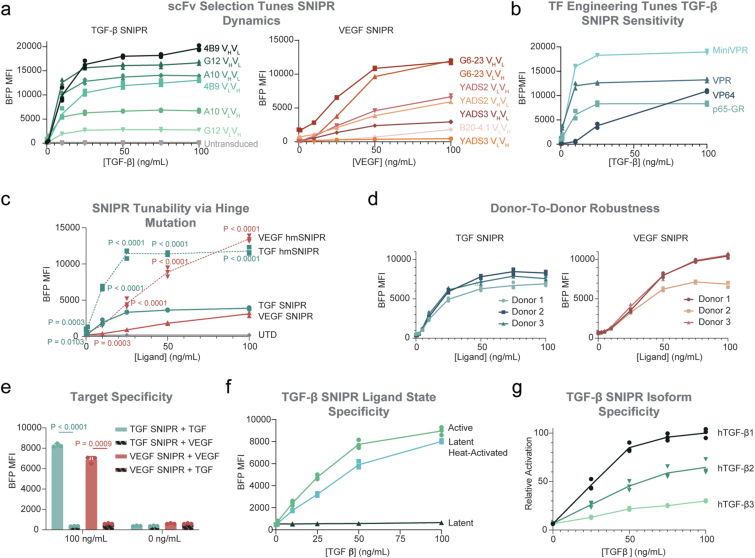
Soluble SNIPR tunability and specificity. **a**, BFP reporter induction in a panel of SNIPR variants bearing scFvs targeted against TGFβ (left) or VEGF (right) in V_H_V_L_ or V_L_V_H_ orientation in primary human T cells. n = 3 (left) and 2 (right) technical replicates. **b**, BFP reporter gene induction of primary human T cells expressing SNIPRs containing a set of unique Transcription Activation Domains (TADs) (n = 2 technical replicates). **c**, BFP reporter induction by conventional or hinge-mutant (hm) TGFβ and VEGF SNIPR or untransduced T cells (n = 3 technical replicates). Statistics represent difference between SNIPR and hmSNIPR variants computed via two-way ANOVA with Šídák’s multiple comparisons test. **d**, BFP reporter activation by TGFβ (left) and VEGF (right) SNIPRs in primary T cells from three different donors (n = 3 technical replicates per donor). **e**, Activation of TGFβ SNIPR and VEGF SNIPR T cells by their matched or mismatched ligands, respectively (n = 3 technical replicates). Statistics calculated using unpaired two-tailed Welch’s t-test. **f**, TGFβ SNIPR activation in primary human T cells using recombinant active TGFβ, recombinant latent TGFβ, or recombinant latent TGFβ re-activated via heat exposure (n = 3 technical replicates). **g**, TGFβ SNIPR activation in primary human T cells using recombinantly expressed human TGFβ1, β2, or β3 protein isoforms (n = 3 technical replicates). Source Data

SNIPRs remain inactive in the absence of ligands but exhibit a robust transcriptional response when membrane-bound ligands are encountered, despite lacking the LNR domains that are thought to be crucial for regulating Notch activation. We hypothesized that SNIPRs are activated through an alternative pathway to the conventional force-induced LNR stretching model^16^, enabling the detection of soluble factors that are inaccessible to receptors of the Notch family. To assess this potential, we first designed SNIPRs with extracellular domains (ECDs) incorporating scFvs derived from a set of antibodies against the broadly tumour-associated cytokines TGFβ (ref. ^5^) and vascular endothelial growth factor-α (VEGF; ref. ^17^). TGFβ is a crucial tumour signalling molecule that is intrinsically tumorigenic, suppressive against tumour-infiltrating immune cells and broadly expressed across a variety of solid cancers^18^. Likewise, VEGF is a pleiotropic tumour-associated signalling molecule that can modulate the immune environment but is best known for its role in stimulating angiogenesis within the tumour^17^. We found that primary human CD3^+^ T cells bearing TGFβ SNIPR driving a BFP reporter circuit signalled after the addition of recombinant activated TGFβ (Fig. 1b). Similarly, a VEGF SNIPR became activated after the titration of recombinant VEGF (Fig. 1c). However, equivalent synNotch receptors did not signal after the addition of the cognate ligands. We showed the tunability of the SNIPR by varying both the scFv identity and orientation (Extended Data Fig. 1a) and the potency of the receptor’s transactivation domain (Extended Data Fig. 1b). We also found that receptor activity can be further improved by replacing the cysteine residue in the hinge domain with a serine residue (Extended Data Fig. 1c). Both classes of SNIPR were strongly activated in response to their respective ligands across several human T cell donors (Extended Data Fig. 1d), and were specific to their designated ligand (Extended Data Fig. 1e). The TGFβ SNIPR is specific to the active versus the latent form of TGFβ (Extended Data Fig. 1f), suggesting that it holds potential for tumour microenvironment detection^19^, and was cross-reactive across human TGFβ isoforms, albeit with a preference for TGFβ1 (Extended Data Fig. 1g). To expand the range of possible soluble targets in disease environments, we developed functional SNIPRs capable of detecting the stromal signalling factor fibroblast growth factor 2 (FGF2) as well as interferon-γ (IFNγ), a key inflammatory cytokine (Fig. 1d). Collectively, these findings indicate that SNIPRs can serve as a versatile receptor architecture for detecting cancer-related and inflammatory soluble signalling molecules.

We next investigated whether the SNIPR system could be adapted for bio-orthogonal cell signalling in T cells. We built SNIPRs incorporating one subunit of a computationally designed ‘LHD’ heterodimer^20^ as the extracellular domain, and evaluated signalling in response to the soluble heterodimeric partner (Fig. 1e). Addition of the cognate subunit resulted in a strong transcriptional response. By contrast, no signalling was observed when the LHD heterodimer was incorporated into the synNotch system. This orthoSNIPR system is designed to be bio-orthogonal, because the LHD subunits do not interact with naturally occurring proteins, and hence enables the creation of ‘private’ channels of communication between engineered cells for precise control over coordinated cellular activity.

## SNIPR mechanism depends on ligand type

**Extended Data Fig. 2:**
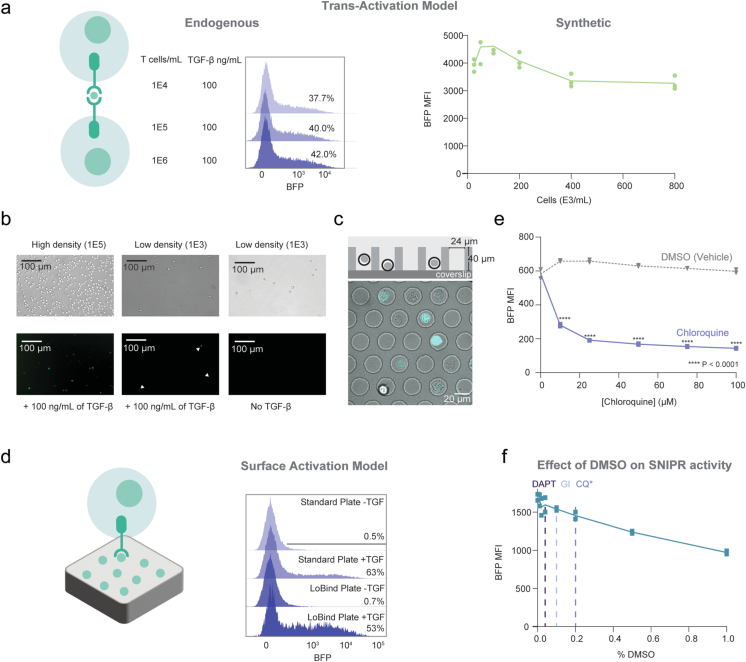
Investigation of soluble SNIPR activation mechanism. **a**, Left, Schematic of a potential model of mechanosensitive soluble SNIPR activation wherein the mechanical force is applied via the simultaneous binding of a dimeric ligand by two SNIPR-expressing cells in trans. Centre: BFP reporter expression histograms depicting a T cell titration assay wherein the TGFβ ligand concentration remains constant while the primary T cells are diluted within the same volume. Measurement obtained 48 h after ligand addition. Right, Activation of an orthoLigand-responsive SNIPR driving a BFP reporter circuit in Jurkat T cells via introduction of orthoLigand C1-active at 500 nM while titrating down the number of receiver cells (n = 3 technical replicates). **b**, Bright-field (top) and fluorescence (bottom) imaging of primary TGFβ SNIPR → NLS-mCitrine circuit human T cells in the presence or absence of recombinant TGFβ1 24 h after ligand exposure. **c**, Confocal maximum intensity projection images of Jurkat T cells containing a TGFβ SNIPR → BFP circuit entrapped in microwells after 24 h of exposure to recombinant TGFβ1. **d**, Left, Schematic of an alternative force-mediated soluble SNIPR activation mechanism wherein mechanical stress is applied via T cell motion while the receptor binds to a putatively soluble ligand which has been immobilized on the cell culture vessel via adsorption. Right, Representative histograms showing TGF SNIPR T cell activation in the presence or absence of 50 ng/mL TGFβ1 in a conventional polystyrene vs. low-binding culture vessel after 24 h of ligand exposure. **e**, BFP reporter expression of TGF SNIPR T cells after addition of 100 ng ml^−1^ TGFβ and the indicated concentration of chloroquine or vehicle control for 24 h (n = 2 technical replicates). Statistics were computed via two-way ANOVA with Šídák’s multiple comparisons test. **f**, BFP reporter expression of TGF SNIPR T cells in the presence of 100 ng ml^−1^ TGFβ and the indicated concentration of DMSO after 24 h of incubation (n = 2 technical replicates). Vertical dashed lines indicate the final DMSO vehicle concentrations for DAPT, GI254023X, and Chloroquine in Fig. 2b (left). The chloroquine and vehicle concentrations used to assay the synthetic receptors were 4-fold lower than that in the endogenous receptor panel. Source Data

The ability of SNIPRs to detect soluble ligands is a new behaviour for Notch-like receptors. Understanding the mechanism of soluble-factor-regulated SNIPR activation has implications for receptor engineering and optimization, and could uncover possibilities for Notch signalling beyond what has been observed in nature. Notch and synNotch signalling is typically considered to be restricted to surface-bound ligands^21^, and previous literature has explicitly demonstrated the inability of a soluble ligand to activate synNotch, in contrast with a membrane-tethered analogue^13,22^. The homodimeric nature of TGFβ and VEGF suggested a model of mechanical cross-activation in *trans*, but SNIPR activation is insensitive to T cell dilution (Extended Data Fig. 2a,b). The ability to induce BFP reporter expression in single cells entrapped in microwells underscores that soluble SNIPR activation does not rely on contact between adjacent cells (Extended Data Fig. 2c). An alternative model proposed that ligands apply force on the receptor by adsorbing to the culture plate, but using low-binding plastic vessels did not abrogate signalling (Extended Data Fig. 2d).

**Fig. 2:**
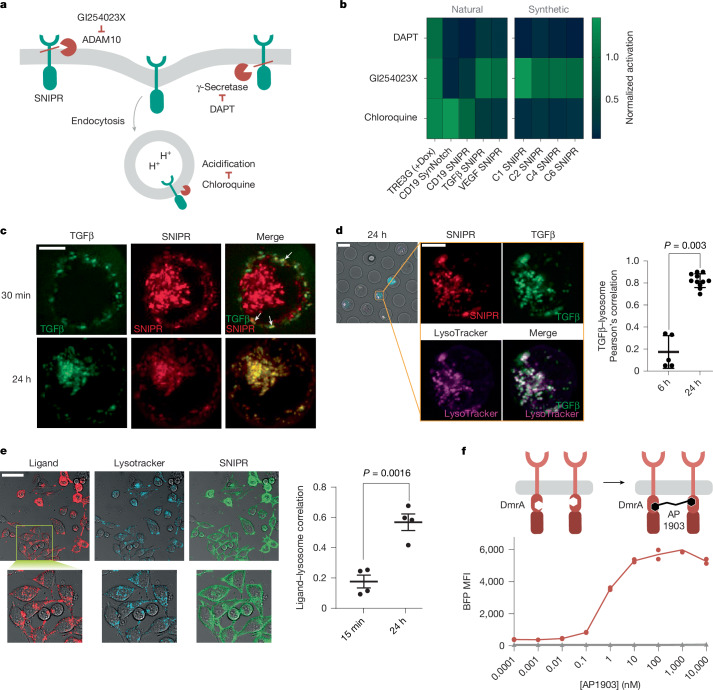
SNIPRs access a distinct activation mechanism. **a**, Schematic of potential activation pathways for soluble or conventional SNIPRs, along with chemical inhibitors to perturb these reactions. **b**, Relative activation of primary human T cells by endogenous ligands, or Jurkat T cells by synthetic ligands, in the presence of the chemical inhibitors described in **a**. T cells expressing soluble or conventional SNIPRs, a conventional synNotch receptor or a proteolysis-independent chemically inducible promoter were selected as a representative panel. Data are mean of *n* = 3 technical replicate measurements after 24 h of incubation normalized to the activation of an inhibitor-free control sample. **c**, Confocal maximum intensity projection images of Jurkat T cells expressing mCherry-fused TGFβ SNIPRs in the presence of recombinant TGFβ1 labelled with AF647. Data are representative of *n* = 25 cells (30 min) or *n* = 27 cells (24 h) across three independent experiments. Scale bar, 5 μm. **d**, Left, confocal maximum intensity projection images of TGFβ SNIPR Jurkat T cells 24 h after exposure to AF647-labelled TGFβ1, costained with LysoTracker. Scale bars, 20 μm (left); 5 μm (right). Right, Pearson’s correlation coefficient between TGFβ1 and LysoTracker. *n* = 10 cells; mean ± s.d. Statistics computed using Welch’s unpaired two-tailed *t*-test. **e**, Left, confocal images of HeLa cells expressing an orthoSNIPR fused to GFP incubated with mCherry-labelled C6-101A orthoLigand and stained with LysoTracker marker for 30 min before imaging. Scale bar, 50 μm. Right, Pearson’s correlation coefficient between C6-101A orthoLigand and LysoTracker. *n* = 4 images per time point with at least 10 cells per image; mean ± s.e.m. Statistics computed using Welch’s unpaired two-tailed *t*-test. **f**, Top, schematic of dimerSNIPR architecture. A DmrA homodimerizing domain was inserted into a TGFβ-responsive SNIPR between the juxtamembrane domain and the transcription factor. The activation assay was performed in the absence of the extracellular TGFβ ligand. Bottom, activation of primary dimerSNIPR T cells by titration of the small molecule AP1903 (*n* = 2 technical replicates). Source Data

Beyond the canonical mechanical model of force-mediated Notch signalling at the plasma membrane, evidence for the role of receptor endocytosis in Notch activation has emerged^23^, and the localization of γ-secretase activity to acidic compartments implies that pH also has a role in some contexts^24^. To probe the behaviour of SNIPRs as they enter the activation pathway, we perturbed each potential step using small-molecule inhibitors (Fig. 2a). All Notch-based receptors, including soluble SNIPRs and CD19-responsive synNotch, are sensitive to γ-secretase inhibition through DAPT, implying a common final trigger for transcription factor release, whereas doxycycline-mediated induction from the control pTRE promoter was insensitive to this perturbation (Fig. 2b). Notably, we found that inhibiting ADAM protease, which is responsible for the initial cleavage event after Notch ligand binding and LNR conformational change, hindered the activation of both the CD19-responsive synNotch and SNIPR, but did not affect the soluble SNIPRs. By contrast, blocking endosomal acidification using chloroquine selectively inhibited the soluble SNIPRs, suggesting a model in which soluble factor binding triggers an endocytic cascade that culminates in proteolysis in the endosome. orthoSNIPRs recapitulated this result in conjunction with a panel of synthetic ligands. Inhibition of the TGFβ SNIPR by chloroquine was dose-dependent (Extended Data Fig. 2e), whereas the effect of the dimethyl sulfoxide (DMSO) vehicle was minimal at the assayed concentrations (Extended Data Fig. 2f). In accordance with the receptor inhibition assay, confocal imaging confirmed that the TGFβ SNIPR colocalizes with a fluorescently labelled TGFβ1 ligand on exposure, and that both receptor and ligand subsequently redistribute to internal compartments (Fig. 2c). In addition, fluorescently labelled TGFβ SNIPR (Fig. 2d) and orthoLigand (Fig. 2e) colocalize with LysoTracker, an endocytic marker, when coincubated.

Despite the data suggesting that SNIPR activation occurs through the endosomal axis, the mechanism by which ligand binding stimulates receptor internalization remained unclear. A potential mechanism is based on the observation that the natural ligand-responsive SNIPRs all bind to dimeric ligands. Receptor oligomerization might induce endocytosis through an avidity-like mechanism in which multiplexed low-affinity binding motifs against the native endocytic machinery cooperatively drive internalization of the clustered complex^25^. We therefore hypothesized that ligand-mediated SNIPR dimerization was responsible for receptor internalization. When the chemically inducible DmrA (FKBP) homodimerization domain^26^ was inserted into the TGFβ SNIPR scaffold (Fig. 2f), the receptor showed strong ligand-independent activation after addition of the AP1903 homodimerizer. Ligand-induced receptor multimerization might serve as the proximal trigger for the endocytic cascade culminating in endosomal transcription factor release and gene expression.

**Fig. 3:**
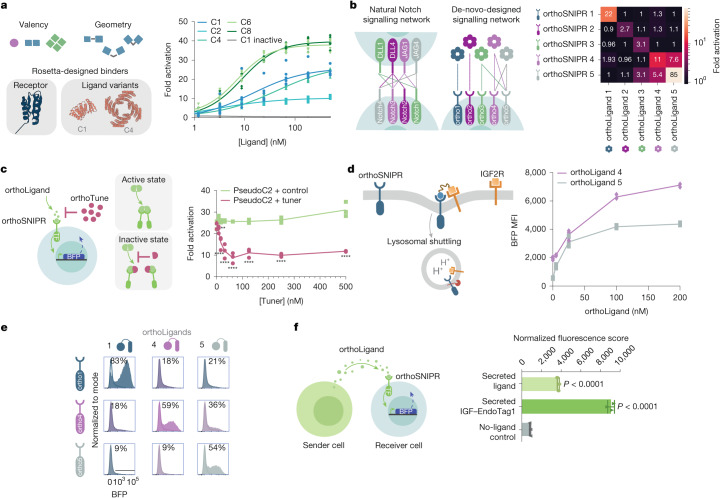
Construction of bio-orthogonal orthoSNIPR signalling systems. **a**, Left, design of a series of synthetic ligands with a range of geometries and valencies. Right, activation of an orthoLigand-responsive SNIPR driving a BFP reporter circuit in Jurkat T cells through the introduction of orthoLigands that vary in their geometry and valency (*n* = 6 technical replicates). **b**, Private and promiscuous signalling channels were created using a set of five heterodimer pairs (left). These pairs were computationally designed to have exclusive binding partners or accommodate promiscuous binding of other heterodimer components. Right, activation of five orthoSNIPRs driving a BFP reporter circuit in Jurkat T cells through the introduction of orthoLigands fused to a C6 scaffolding backbone. Ligands were added at 500 nM concentration (data are mean of *n* = 3 technical replicates). **c**, Left, schematic of a modulation strategy for orthoLigand-responsive SNIPRs through the introduction of orthoTuners generating a pseudo-C2 structure by homodimerization. Right, BFP reporter activation in Jurkat T cells after the addition of C2 orthoLigand and the orthoTuner dimerization disrupter (*n* = 3 technical replicates). Statistics calculated using two-way ANOVA with Šídák’s multiple comparisons test. **P = 0.0077; ****P < 0.0001. **d**, Enhancing orthoLigands by forced lysosomal shuttling. Left, schematic of EndoTag-mediated soluble SNIPR activation. Right, activation of orthoSNIPR4 and orthoSNIPR5 in primary human T cells using recombinant orthoLigand–EndoTag 4 and 5 (respectively) 24 h after ligand exposure (*n* = 3 technical replicates). **e**, Activation of three different orthoSNIPRs in primary human T cells using their cognate and mismatched EndoTagged orthoLigands (200 nM, 24 h ligand exposure, representative of *n* = 3 technical replicates). **f**, Left, schematic of autonomous cell-to-cell communication through orthoLigand secretion. Right, activation of an orthoLigand-responsive SNIPR driving a BFP reporter circuit in Jurkat T cells through the introduction of orthoLigands secreted from sender HeLa cells (data are mean ± s.e.m. of *n* = 3 technical replicates). Statistics computed using one-way ANOVA with Dunnett’s multiple comparisons test. Source Data

**Extended Data Fig. 3:**
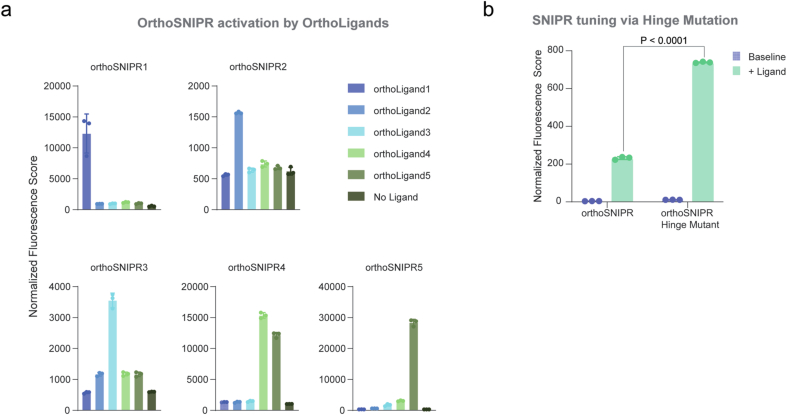
OrthoSNIPR activation and tuning. **a**, Individual activation analysis of the 5 × 5 receptor ligand screen shown in Fig. 3c. n = 3 technical replicates. Error bars represent SEM. **b**, Activation of Jurkat T cells expressing orthoSNIPR and a corresponding variant bearing the hmSNIPR hinge cysteine substitution using orthoLigand C1-active at 200 nM (n = 3 technical replicates; error bars represent SEM). Statistics were computed using Welch’s unpaired *t*-test. Source Data

We next investigated the tunability of orthoSNIPR signalling. We generated ligands with the same binding domain but differing valency and geometry by fusing them to a series of designed oligomeric scaffolds^27^ (Fig. 3a). These ligands generate a range of signalling strengths, as reflected in both the half-maximum effective concentration (EC_50_) and the maximum effective concentration (*E*_max_), with higher-valency ligands generally having increased sensitivity and cooperativity and a maximum activation of up to 40-fold over the baseline level. To enable the creation of bio-orthogonal communication networks, we generated a series of orthoSNIPR receptor–ligand pairs using five different synthetic heterodimer pairs. As above, we used one subunit of each heterodimer as the extracellular domain of the SNIPR, and the second subunit was fused to a designed hexameric scaffold conferring robust signalling. To maximize the versatility of the signalling networks that could be generated from these synthetic receptors, we chose three heterodimer pairs that previous characterization with purified proteins showed are completely orthogonal, and two heterodimers that cross-interact; the former enable insulated communication pathways, and the latter enable combinatorial modulation, as observed with BMP signalling^28^ (Fig. 3b and Extended Data Fig. 3a). As predicted from the biochemical properties of the binding domains, three of the receptors are completely orthogonal and respond only to their ligands, whereas the other two recapitulate the more relaxed specificity of the parent designs. Such cross-reactive components enable the design of complex networks with fewer parts than would be possible with fully orthogonal receptors, allowing more information to be encoded in therapeutic cells by payload-limited gene-delivery vectors. Thus, the orthoSNIPR system can be readily expanded beyond one receptor to form the basis of a complex communication network that relies on private and promiscuous signals to process and respond to their environments and execute user-defined functions in therapy, development or homeostasis.

The orthoSNIPR platform offers several avenues for signal tuning in addition to modulating ligand valency. Similar to the TGFβ and VEGF SNIPRs, orthoSNIPR signalling output increases when the hinge cysteine is mutated to a serine residue (Extended Data Fig. 3b). A more dynamic approach uses engineered signalling pathways that are conditional on the presence of additional factors, which could have considerable therapeutic utility. For example, if CAR T cells detect a signal from other engineered cells indicating that they are in the wrong environment, or if patients experience symptoms of cytokine release syndrome, off switches or damper switches could mitigate toxicity. To enable such conditional signalling, we designed a weakly homomeric ligand that strongly induces receptor signalling unless another engineered factor, orthoTuner, is present in the environment. orthoTuner has a higher affinity for the subunits of the homodimeric ligand than they have for each other, and hence forms heterodimers that contain only one receptor-interacting LHD module (Fig. 3c). Adding orthoTuner inhibits signalling in a dose-dependent manner. This conditionality enables the strength of orthoSNIPR signalling to be modulated by external inputs or communication between engineered cells.

Deciphering the mechanism of soluble SNIPR activation suggested an approach to enhance the signal strength of orthogonal output by promoting its endocytosis. Tethering orthoLigands to binders against lysosomal shuttling proteins (EndoTags) promotes the internalization of SNIPR and the activation of its payload (see companion manuscript^29^). EndoTagged orthoLigands efficiently activate their cognate SNIPRs in primary T cells (Fig. 3d,e). We extended our work on the orthoSNIPR system to demonstrate autonomous cell signalling with cell-derived rather than exogenous ligands. To this end, we engineered a ‘sender’ cell that secretes the ligand. When the ‘receiver’ Jurkat T cells expressing the cognate receptor were cultured in the presence of the medium conditioned by the sender cells, we observed signalling in response to the secreted ligand (Fig. 3f). We chose to isolate the sender cells from the receiver cell to avoid cell-membrane-based transactivation and guarantee that any response is due to the soluble ligand. Sender cells secreting isolated orthoLigand activated SNIPR-bearing receiver cells, and secreted EndoTagged orthoLigand augmented the response. Our experiment shows that with the orthoSNIPR communication system, cells can autonomously communicate and share information in a channel that is completely private and biorthogonal.

## Therapeutic soluble SNIPR→CAR circuits

**Fig. 4:**
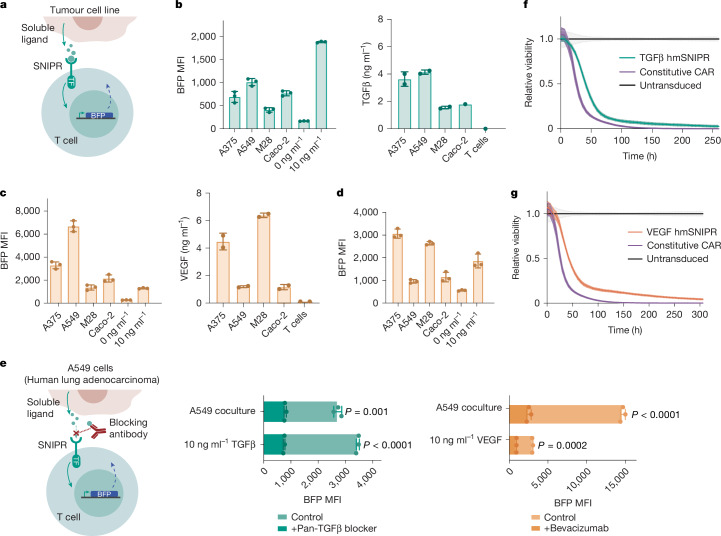
In vitro evaluation of engineered cell–cell communication through soluble ligands. **a**, Schematic of in vitro activation experiment. Cocultured cancer cell lines secrete endogenous soluble factors that activate the cognate SNIPR, driving expression of the downstream BFP reporter. **b**, Left, in vitro activation of primary human T cells bearing TGFβ SNIPR in coculture with the respective cancer cell line (*n* = 3 technical replicates; mean ± s.d.). Right, TGFβ1 secretion of cancer cell lines, measured by ELISA (*n* = 2 technical replicates). Error bars represent s.d. **c**, Left, in vitro activation of primary human T cells bearing VEGF SNIPR in coculture with the respective cancer cell line (*n* = 3 technical replicates; mean ± s.d.). Right, VEGF secretion of cancer cell lines measured by ELISA (*n* = 2 technical replicates). **d**, In vitro activation of primary human T cells bearing VEGF SNIPR in transwell cell culture with the respective cancer cell line (*n* = 3 technical replicates; mean ± s.d.). **e**, Left, schematic of ligand blocking coculture experiment. BFP reporter activation of primary human TGFβ (middle) or VEGF (right) SNIPR T cells in coculture with A549 target cells, or with the addition of recombinant ligand. Activation was performed with and without a simultaneously administered blocking antibody and measured 24 h after treatment. Data are mean of *n* = 3 technical replicates ± s.d. Statistics computed using Welch’s unpaired two-tailed *t*-test. **f**, Relative killing efficacy of TGFβ SNIPR→CAR or constitutive HER2 CAR T cells targeting A549^RFP-NLS^ cells, measured by Incucyte live-cell imaging. hmSNIPR, hinge mutant SNIPR. **g**, Incucyte imaging of VEGF SNIPR→CAR or constitutive HER2 CAR T cells targeting A549^RFP-NLS^ cells. Source Data

**Extended Data Fig. 4:**
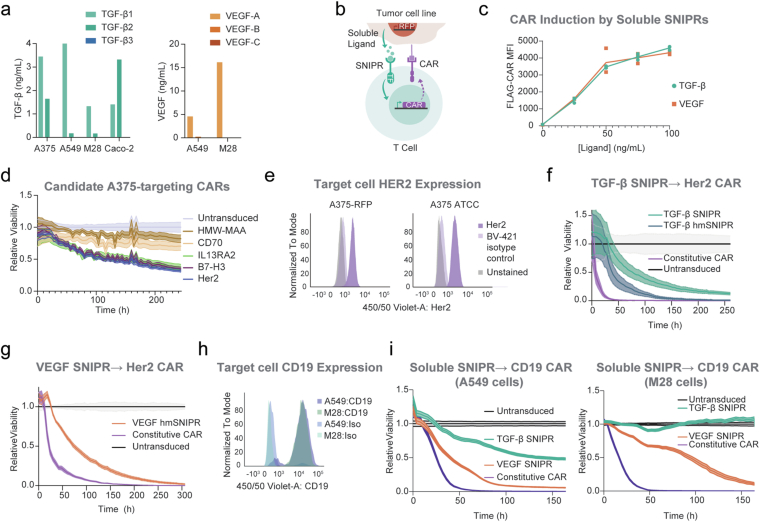
Soluble antigen sensors can improve CAR-T therapy. **a**, Quantification of TGFβ and VEGF isoform secretion from cancer cell lines using Multiplexing Laser Bead assay. **b**, Schematic of Incucyte cytotoxicity assay; RFP-labelled tumour cell lines induce CAR expression in soluble SNIPR → CAR T cells by secreting the cognate SNIPR ligand. **c**, Induction of a Flag-tagged Her2 CAR by TGF or VEGF SNIPR T cells using titrated recombinant ligand (n = 3 technical replicates). **d**, Incucyte imaging of CD8^+^ T cells bearing a panel of TGF SNIPR→CAR circuits cocultured with A375^RFP-NLS^ cells. **e**, Quantification of Her2 expression on A375^RFP-NLS^ and early passage A375 cells sourced from ATCC. **f**, Incucyte cytotoxicity analysis of TGFβ SNIPR → Her2 CAR circuit variants, constitutive Her2 CAR, and untransduced T cells. **g**, Incucyte cytotoxicity assay of VEGF SNIPR → Her2 CAR, constitutive CAR, and untransduced T cells. **h**, Comparison of engineered CD19 expression on A549 and M28 target cells. **i**, Incucyte viability analysis of A549 (left) and M28 (right) cells ectopically expressing CD19 in coculture with TGFβ or VEGF SNIPR→CD19 CAR, constitutive CD19 CAR or untransduced T cells. Source Data

To show that SNIPRs could detect the physiological production of endogenous soluble factors rather than overexpressed or supplemented recombinant ligands, we assayed SNIPR→BFP circuit T cells in coculture with a panel of potential target human tumour cell lines (Fig. 4a). The activation of BFP expression in TGF SNIPR T cells generally scales with the production of TGFβ1 (Fig. 4b) across tumour cell lines, as determined by enzyme-linked immunosorbent assay (ELISA). The disproportionate response to Caco-2 can be reconciled by Luminex analysis, revealing the coproduction of TGFβ2 alongside the primary TGFβ1 isoform **(**Extended Data Fig. 4a). Activation of VEGF SNIPR T cells in direct coculture with target cells shows a less clear correlation with the measured VEGF concentration, with A549 cells stimulating disproportionate BFP expression (Fig. 4c). Luminex analysis reveals that VEGFα is the sole variant of VEGF produced (Extended Data Fig. 4a), ruling out cross-homologue reactivity as the cause. Notably, preventing direct cell–cell contact using a transwell cell-culture system reconciles the BFP expression pattern with the measured ligand secretion (Fig. 4d), indicating that the activity of A549 is increased in a direct coculture setting. Nonetheless, the specificity of activation was confirmed by inhibiting SNIPR activation through TGF- and VEGF-blocking antibodies (Fig. 4e), which effectively blunted BFP expression, including in coculture with A549. The heightened activation potential of the A549 cell line could be due to surface display of VEGF, or to other undetermined mechanisms that rely on cell contact.

Soluble antigen sensors could improve the safety and efficacy of CAR T cell therapy by selectively restricting CAR expression in the tumour, imparting asynchronous IF→THEN combinatorial logic and enhancing the specificity of potentially promiscuous CARs. A receptor intended for therapeutic use must strike a balance between low basal signalling to avoid off-target toxicity and robust activation to drive a sufficient level of CAR transgene expression for effective cancer-cell killing. This is particularly challenging given the lower sensitivity of CARs compared with their natural T cell receptor counterparts^30^. We sought to construct circuits in which soluble SNIPR-primed activation drives the expression of a CAR capable of addressing solid tumour antigens (Extended Data Fig. 4b). We verified that, similarly to BFP, the CAR payload is expressed by SNIPR→CAR T cells in a dose-dependent manner (Extended Data Fig. 4c). To determine the optimal CAR payload for targeting A375 melanoma cells, which have previously been implicated in TGF-mediated CAR T cell suppression^31^, we constructed TGFβ SNIPR→CAR circuits bearing a panel of CARs against potential endogenous A375 antigens, and assayed their in vitro killing efficacy using a live-cell imaging assay (Extended Data Fig. 4d). Of the variants tested, a HER2-specific CAR was the most efficacious and was therefore selected for subsequent experiments. Although this result was surprising, in light of literature suggesting a dearth of HER2 expression in A375 cells^32^, the presence of this antigen was confirmed in RFP-expressing target cells used for live-cell killing assays as well in the unmodified cell line (Extended Data Fig. 4e). We then showed that both TGFβ and VEGF-responsive SNIPR circuits driving the HER2 CAR exhibit robust killing of both A549 lung adenocarcinoma cells (Fig. 4f,g) and A375 cells (Extended Data Fig. 4f,g). Consistent with previous work, SNIPR→CAR circuits displayed slower killing kinetics than did constitutively expressed CAR, owing to the temporal delay in CAR expression subsequent to primary ligand encounter^15^. To verify the target specificity of these circuits, we constructed A549 and M28 cells overexpressing matched levels of CD19 (Extended Data Fig. 4h). As predicted by reporter gene induction in coculture, the TGFβ SNIPR shows specificity for A549, whereas the VEGF SNIPR shows preferential A549 targeting (Extended Data Fig. 4i).

**Fig. 5:**
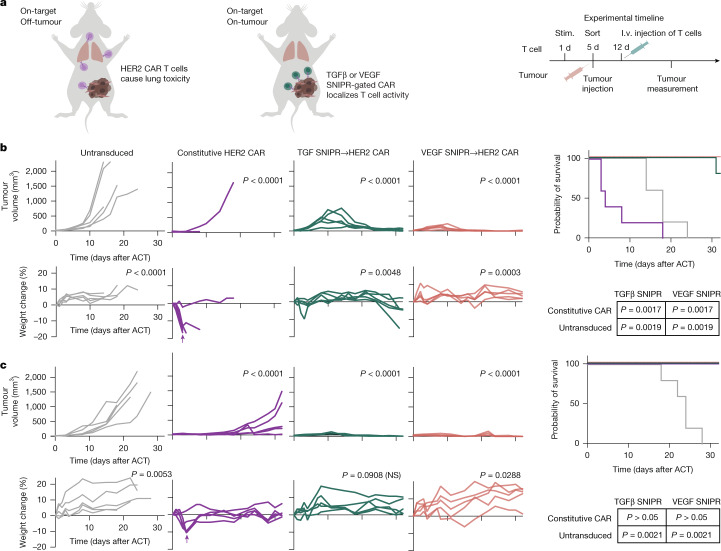
In vivo anti-tumour efficacy of soluble SNIPR→CAR T cells. **a**, Schematic of in vivo CAR efficacy and toxicity study. The cross-reactive CAR has previously been shown to induce toxicity, most prominently displayed as a weight-loss phenotype, owing to on-target and off-tumour activity targeting mainly lung tissue. NSG mice were implanted subcutaneously with A549 lung carcinoma or A375 melanoma cells. After 7 days, mice were adoptively transferred (i.v.; intravenously) with primary human CD3^+^ T cells bearing soluble SNIPRs driving a mouse–human cross-reactive HER2 CAR, constitutively expressed HER2 CAR or untransduced T cells. Stim., stimulated. **b**, Tumour volume (top), weight change (bottom), and survival (right) of A549 xenografted mice. Tumour volume statistics analysed using one-way ANOVA with Dunnett’s correction comparing the area under the curve versus the untransduced control group over the course of the experiment. Weight loss statistics computed by one-way ANOVA with Dunnett’s correction comparing mouse weights versus constitutive CAR on the day of peak weight loss (indicated by an arrow). Survival statistics calculated using pairwise log-rank test with Bonferroni correction factor comparing SNIPR→CAR T cells with constitutive CAR and untransduced control groups. ACT, adoptive cell transfer. **c**, Activity of SNIPR circuits driving the ^tr^DARPin HER2 CAR in A375 melanoma xenografted mice. Data presented analogously to **b**. Tumour volume statistics analysed using one-way ANOVA with Dunnett’s correction comparing the area under the curve versus the untransduced control group over the course of the experiment. Weight loss statistics computed by one-way ANOVA with Dunnett’s correction comparing mouse weights versus constitutive CAR on the day of peak weight loss (indicated by an arrow). Survival statistics calculated using pairwise log-rank test with Bonferroni correction factor comparing SNIPR→CAR T cells with constitutive CAR and untransduced control groups. NS, not significant. Source Data

**Extended Data Fig. 5:**
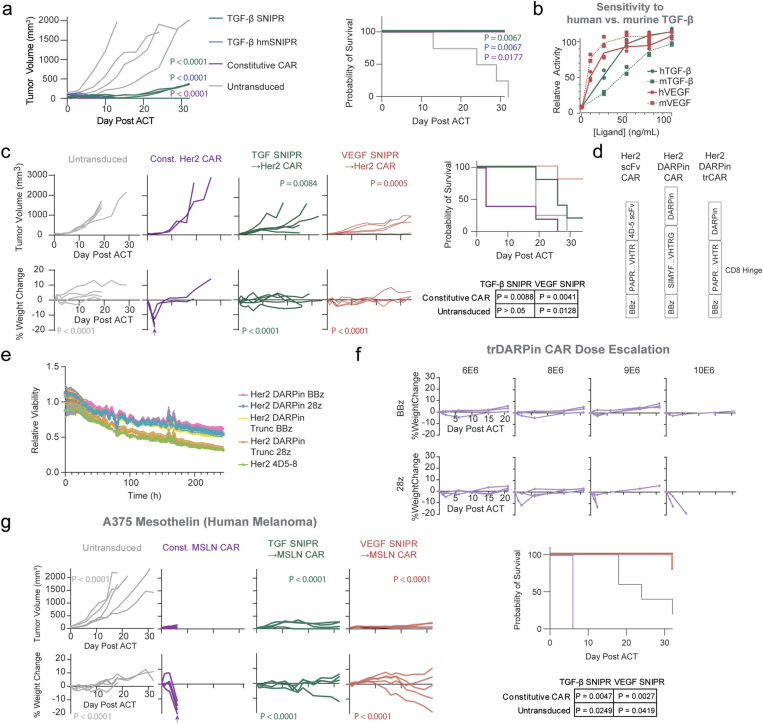
In vivo performance of SNIPR–CAR circuits. **a**, Tumour volume (left) and Kaplan-Meyer survival plots (right) of TGFβ SNIPR, hinge-mutant SNIPR, or constitutive 4D5 anti-Her2 CAR T cells versus untransduced control T cells. Tumour volume statistics compare cumulative area under curve versus untransduced control using one-way ANOVA with Dunnett’s correction. Survival statistics versus untransduced control were calculated using pairwise Log-Rank test with Bonferroni correction factor. **b**, Activation of TGFβ (left) and VEGF (right) SNIPR → BFP T cells using recombinant human and mouse ligands (n = 3 technical replicates). **c**, Tumour growth (top), weight change (bottom), and survival (right) of NSG mice bearing A375 xenografts treated with untransduced, constitutive, or SNIPR → Her2 DARPin CAR T cells. Statistics are presented analogously to those shown in Fig. 5c,d). **d**, Schematic of CD8 hinge sequence variation in the original 4D5 CAR, original DARPin CAR, and matched truncated DARPin CAR. **e**, Incucyte comparison of cytotoxic potential of Her2 DARPin CARs in original or truncated CD8 Hinge architectures and bearing CD28 vs. 4-1BB costimulatory domains. **f**, Weight loss phenotype of tumour-free NSG mice injected with constitutive ^tr^DARPin Her2 CAR T cells bearing 4-1BB (top) or CD28 (bottom) costimulatory domains. **g**, In vivo evaluation of a constitutive or SNIPR-gated mouse/human cross-reactive Mesothelin CAR in an NSG xenograft model. Tumour volume (top), weight change (bottom), and overall mouse survival (right) are shown. Statistics are presented analogously to those shown in Fig. 5c. Source Data

We next evaluated the in vivo performance of SNIPR→CAR circuits. Mice bearing subcutaneously embedded A375 melanoma tumours underwent adoptive T cell transfer seven days after tumour seeding. TGFβ SNIPR→HER2 CAR T cells significantly slowed tumour growth relative to untransduced control T cells, whereas SNIPRs bearing the activating hinge mutation eliminated the tumours with an efficacy similar to that of the constitutive CAR (Extended Data Fig. 5a). To assess the ability of soluble SNIPRs to improve the therapeutic window of CARs that are prone to off-tumour toxicity, we replaced the human-specific 4D5 HER2 CAR with a previously described mouse–human cross-reactive DARPin-based variant^33^. Previous work showed that constitutive DARPin CAR T cells administered to NSG mice result in toxicity and weight loss, owing to on-target and off-tumour toxicity against low levels of HER2 expression in lung tissue^34^—consistent with the fatal pulmonary toxicity reported in an early clinical trial of human HER2-targeting CARs^35^. SNIPR→CAR circuits are expected to enhance the safety of CARs with such promiscuous activity by confining their expression to the tumour (Fig. 5a). The ability of TGFβ and VEGF SNIPRs to activate in response to both human and mouse target ligands (Extended Data Fig. 5b) further enhances the clinical relevance of this model, although TGFβ and VEGF expression in immunocompromised NSG mice might not recapitulate that of syngeneic models.

We first evaluated the performance of our SNIPR→DARPin CAR circuits in an A549 adenocarcinoma xenograft model. Consistent with the previously published data, mice injected with constitutive DARPin CAR T cells underwent rapid weight loss to the humane euthanasia threshold (Fig. 5b). By contrast, mice that received the SNIPR→CAR circuits maintained a healthy weight and were able to robustly control tumour growth, emphasizing the potential of this approach for generating potent cell-based therapies with improved safety profiles. We then sought to validate our circuits in an A375 melanoma xenograft model. Despite retarding tumour growth, the SNIPR circuit T cells did not clear the tumours, which suggests that A375 xenografts are more resistant to T cell cytotoxicity (Extended Data Fig. 5c). An in vitro cytotoxicity analysis revealed that the DARPin CAR, constructed as reported with an extended CD8 hinge domain, is less potent than the original 4D5-based version, but we found that a modified construct bearing a truncated hinge (DARPin ^tr^CAR), matching that in the 4D5 CAR, partially restored efficacy (Extended Data Fig. 5d,e). Notably, we also found that this truncation reduced the in vivo toxicity of the constitutive CAR T cells, necessitating the installation of the CD28 costimulatory domain and injection of a higher dose to recapitulate weight loss (Extended Data Fig. 5f). We then evaluated the performance of the SNIPR→^tr^CARs in the A375 xenograft model, increasing the tumour dosage proportionally to the T cells. The TGFβ and VEGF SNIPR→^tr^CAR circuits were well tolerated and highly efficacious, whereas constitutive DARPin ^tr^CAR T cells rapidly induced weight loss within several days of infusion, albeit not to the euthanasia threshold, and then were unable to control tumour outgrowth (Fig. 5c). To test an orthogonal toxicity model, we also evaluated our constructs using a cross-reactive anti-mesothelin nanobody-based CAR payload^36^, with A375 tumour cells ectopically expressing mesothelin. Similarly to the cross-reactive HER2 CARs, the SNIPRs significantly increased the therapeutic window of this potent and highly toxic CAR (Extended Data Fig. 5g).

## Discussion

The SNIPR architecture satisfies the demands of high-performance soluble factor sensing. SNIPRs bearing ligand-binding domains that target cancer-associated factors show robust activation after the titration of recombinant ligands, and are capable of driving potent therapeutic responses at the site of disease, mitigating the toxicity potential of cell therapies. The ability of SNIPRs to recognize engineered orthogonal ligands expands their potential capabilities beyond natural cues and unidirectional signalling. Immune cells bearing orthoSNIPRs could coordinate to establish signalling feedback loops to more precisely pinpoint tumour coordinates in the body, and different immune cell types bearing their own receptors and payloads could interact to activate specific programs at the optimal times during treatment stages. Customizing orthoSNIPR signalling using privileged or promiscuous signalling channels enables the fine-tuning of engineered cell behaviour, and the capability to sense and secrete endogenous signalling factors provides an interface with the local host immune environment.

Some aspects of soluble SNIPR biology remain open to further investigation. The ability to increase soluble SNIPR activity with a cysteine substitution in the hinge domain suggests that additional mechanisms are available to fine-tune the response profile of these receptors. In addition, the high response of the VEGF SNIPR T cells to A549 cells, which seem to secrete relatively little VEGF, suggests that the receptor may be triggered in response to a combination of ligand states consisting of both fully soluble and membrane-bound ligand. Unravelling this behaviour might provide further avenues to shape SNIPR T cell responses to the surrounding microenvironment.

Overall, soluble SNIPRs provide a versatile tool for therapeutic bioengineering and other disciplines in biology. The customizability of these receptors could enable them to sense morphogen gradients established by proteins such as NGF and the BMP family during embryonic development, or to report on the local immune state in the context of cancer, autoimmunity and infectious disease. These receptors are also suitable for use in developing complex high-order biocomputational circuits, owing to their programmability through synthetic ligands and their compatibility with modular payloads. Their compact genetic footprint might permit the development of multi-receptor circuits that integrate various soluble or cell-surface inputs for precise localization of biological activity^37^. Soluble SNIPRs expand our ability to precisely control cells for therapeutic purposes and basic biology applications to study cellular interactions.

## Methods

### Plasmid assembly

Plasmids were derived from previously reported constructs, with receptor vectors originating in pHR_PGK (Addgene 79120) and reporter constructs cloned into pHR_Gal4UAS_PGK_mCherry (Addgene 79124). All constructs reported in this work were generated using NEBuilder HiFi DNA Assembly (NEB E2621). A mapping of which constructs correspond to which figures is shown in Supplementary Table 1. Sequences are listed in Supplementary Table 2. The origins of new components described in this manuscript are listed in Supplementary Table 3.

### Cell culture and lentiviral production

Lenti-X 293T cells (Clontech 632180) were cultured in Dulbecco’s modified Eagle’s medium (DMEM) supplemented with 10% FBS (Millipore Sigma), 1× sodium pyruvate (Millipore Sigma) and 50 U ml^−1^ penicillin–streptomycin (MP Biochemicals). A375 cells expressing nuclear-localized RFP were a gift from the A. Marson laboratory at UCSF. A549 cells (CCLZR013) were obtained from the UCSF Cell and Genome Engineering Core. A375 and A549 cell lines were grown in 293T medium. Caco-2 cells were obtained from ATCC (HTB-37) and grown in DMEM (Gibco 11965092) supplemented with 20% FBS and 50 U ml^−1^ penicillin–streptomycin. M28 cells were obtained from the B. Gerwin laboratory at the National Cancer Institute and grown in RPMI 1640 (Gibco) with 10% FBS, 50 U ml^−1^ penicillin–streptomycin and 1× GlutaMAX (Gibco). Cells obtained from ATCC or from the UCSF Cell and Genome Engineering Core were authenticated by the supplier. All cell lines were tested for mycoplasma (ATCC 30-1012K). For viral production, 1E6 Lenti-X 293T cells were seeded in a six-well vessel at 1 × 10^6^ per well in 2.5 ml of 293T medium. One day after seeding, cells were transfected with a packaging mix consisting of 1.5 μg transgene expression vector, 1.34 μg pCMVdR8.91 and 0.17 μg pMD2.G, using TransIT-Lenti Transfection Reagent (Mirus) (3 μl of the reagent per 1 μg of total DNA). Two days after transfection, viral supernatants were collected by centrifugation and used immediately for transduction.

For Jurkat experiments, HEK293T cells were first cultured in DMEM (Gibco) supplemented with 10% FCII (Gibco) and 50 U ml^−1^ penicillin–streptomycin (Gibco), then with ‘induction’ DMEM medium supplemented with 10% FCII, 10 mM sodium butyrate and 50 U ml^−1^ penicillin–streptomycin, and later with ‘viral’ DMEM medium supplemented with 10% FCII, 0.1 mM HEPES, 1× GlutaMAX, 1× MEM non-essential amino acids and 50 U ml^−1^ penicillin–streptomycin. Jurkat T cells were cultured in RPMI 1640 supplemented with 1× GlutaMAX (Gibco), 10% FCII and 50 U ml^−1^ penicillin–streptomycin. For lentivirus production, 293T cells were seeded at 400,000 cells per ml in either a 12-well vessel or a T75 plate and grown to 90% confluency. Then, 22 μg expression vector was mixed with 22 μg pCMV and 4.5 μg pVSV-G (for 12-well transfections, these microgram quantities of DNA were divided by 4). This DNA mixture was then packaged using PEImax (Thermo Fisher Scientific) and 1× Opti-MEM (Gibco) and added to the medium of the 293T cells. Then, 12–17 h later the medium was removed from these cells and replaced with ‘induction’ DMEM medium containing 10 mM sodium pyruvate. After incubating for 6–8 h, the ‘induction’ medium was replaced with ‘viral’ medium. Two days later, viral supernatants were collected using Lenti-X Concentrator (Takara), concentrated by centrifugation and used immediately for transduction.

### Primary human T cell culture

T cells were isolated from anonymized donor blood after apheresis (Vitalant) in bulk CD3^+^ format through positive selection (Stem Cell 17851). For Incucyte experiments, CD8^+^ populations were isolated (Stem Cell 15063). Use of donor material was approved by the UCSF Institutional Review Board. After isolation, T cells were frozen in liquid nitrogen in RPMI 1640 (Thermo Fisher Scientific 11875093) supplemented with 20% human AB serum (Valley Biomedical HP1022) and 10% DMSO. For experiments, T cells were thawed at 37 °C, washed and cultured in human T cell medium consisting of X-VIVO15 (Lonza 04-418Q) with 5% human AB serum, 10 mM *N*-acetyl l-cysteine (Sigma-Aldrich A9165) neutralized with 1 M NaOH (Sigma-Aldrich S2770) and supplemented with 30 units per ml IL-2 (NCI BRB Preclinical Repository). One day after thaw, T cells were stimulated with washed Dynabeads (Thermo Fisher Scientific 11132D) using a 1:3 cell:bead ratio. On the following day, untitred lentiviral supernatant was added at a 1:1 total volume ratio. After 24 h of infection, cells were gently pelleted (400*g* for 5 min) and depleted medium was exchanged for fresh complete medium. After three days of subsequent expansion, Dynabeads were removed by magnetic separation and cells were sorted (Beckton Dickinson FACSAria II) for populations expressing both epitope-tagged receptors and constitutive fluorophore-expressing reporter circuits (where applicable). Experiments typically commenced 4 to 7 days after sort. All experiments were performed in complete human T cell medium other than Incucyte assays, which were performed in RPMI–10% FBS.

### Flow cytometry and sorting

For sorting, T cells were pelleted and resuspended in 100 μl PBS with 2% FBS and antibody at a 1:100 ratio. After 20–30 min of staining at room temperature, cells were pelleted and resuspended in PBS with 2% FBS and kept on ice until sorting. For flow cytometry measuring a fluorescent reporter gene, cells were directly analysed and gated on scatter (FSC-A versus SSC-A), single cells (FSC-H versus FSC-A) and the constitutive co-fluorophore expressed by the reporter construct (Supplementary Fig. 1a). For cytometry experiments involving immunohistochemistry, cells were washed once in PBS before staining for 20–30 min at room temperature, and then washed three times before analysis. The antibodies used are listed in Supplementary Table 4.

### In vitro SNIPR activation

Recombinant soluble factors were gently resuspended in their respective manufacturer-recommended reconstitution buffers and frozen at −80 °C as single-use aliquots. After thawing at room temperature, proteins were diluted in T cell medium to the appropriate concentration and either added directly to the T cells in 96-well plates at a 1:100 dilution, or pre-diluted in medium to 2× final concentration and added in a 1:1 ratio. Cells were mixed briefly and incubated at 37 °C until reporter expression was assayed by flow cytometry (BD FACSymphony X50 SORP or LSR II SORP). The data shown represent 72 h of ligand exposure unless otherwise indicated. Representative gating is shown in Supplementary Fig. 1b. The recombinant soluble factors used in experiments are listed in Supplementary Table 5, and inhibitors are shown in Supplementary Table 6. Where unspecified, TGFβ refers to human TGFβ1 and VEGF refers to human VEGFα. Activation of latent human TGFβ1 was performed by incubation at 80 °C for 5 min as described previously^38^. For the TRE3G promoter, doxycycline hyclate (Abcam ab141091) was added as the ligand analogue reagent. For chemical inhibitor experiments, DAPT, GI254023X and chloroquine were used at a working concentration of 10 μM, 10 μM and 25–100 μM, respectively. The blocking antibodies, pan-TGFβ blocker (R&D MAB1835) and bevacizumab (Selleck, A2006) were used at a concentration of 0.17 μg ml^−1^ and 1.4 μg ml^−1^, respectively. Chemical inhibitors and blocking antibodies were added immediately before ligands.

For Jurkat experiments, Jurkat T cells were collected and resuspended at 400,000 cells per ml in fresh RPMI medium. De-novo-designed orthoLigands were diluted to the appropriate concentrations in 1× PBS and were added to freshly washed cells at a 1:10 dilution (20 μl into 200 μl) in 96-well plates. Assay plates were incubated at 37 °C for 17–24 h and then analysed using flow cytometry. The orthoLigands and inhibitors used in these experiments are also listed in Supplementary Table 5 and Supplementary Table 6, respectively.

### Microwell fabrication

Microwells were prepared as previously described^39^. In brief, a master SU8 mould with periodic arrays of microwells (24 µm × 40 µm diameter × depth, 10 µm well-to-well spacing) was fabricated on a silicon wafer by photolithography. The master mould was then passivated by O_2_-plasma treatment followed by trichloro(1H,1H,2H,2H-perfluorooctyl)silane (Sigma-Aldrich) vapour deposition under vacuum. Polydimethylsiloxane (PDMS) was cast onto the wafer and cured to create a negative of the master mould. After carefully removing PDMS blocks from the wafer, a circular PDMS stamp (4 mm in diameter) was cut and placed on the coverslip (no. 1.5) of a glass-bottomed dish (MatTek). A drop of pre-polymer solution (BIO133, My Polymers) was added to one side of the PDMS stamp, facilitating gap-filling between the substrate and the stamp by capillarity. After curing with UV exposure, the PDMS stamp was removed carefully. The final microwell array pattern was treated with pluronic acid F127 (Sigma-Aldrich) overnight to block nonspecific binding of soluble ligands to the microwells.

### Protein expression and purification

orthoSNIPR ligands were expressed in *Escherichia coli* BL21 (NEB). In brief, the DNA fragments encoding the design sequences were assembled into pET-29 vectors by Gibson assembly and further transformed into the BL21 strain with heat shock. Protein expression was induced by the autoinduction system and proteins were purified with immobilized metal affinity chromatography (IMAC). The elutions were further purified by FPLC SEC using Superdex 75 10/300 GL or Superdex 10/300 200 columns (GE Healthcare). Protein concentrations were determined by NanoDrop (Thermo Fisher Scientific) and normalized by extinction coefficients. Proteins were diluted to the appropriate concentrations in 1× PBS and applied to cell culture medium using 1:100 dilutions.

### Confocal microscopy

HeLa cells were engineered to express the SNIPR receptor with a cleavable GFP domain in place of a transcription factor. These cells were seeded in 18-well glass-bottomed µ-Slides (Ibidi 81817) at a density of 15,000 per well. mCherry-fused SNIPR ligands were incubated with the cultured cells for 15 min, 3 h, 6 h or 24 h, and LysoTracker (Thermo Fisher Scientific L7526) was added 30 min before imaging. Cells were washed three times in PBS and imaging was performed immediately. Confocal laser scanning microscopy was performed on a Nikon A1R HD25 system equipped with a LU-N4 laser unit (Lasers used: 488 nm, 561 nm and 640 nm). Data were acquired using a 20×, NA 0.75, WD 1.00 mm air objective (Plan Apochromat Lambda) in combination with one multialkaline (EM 650 LP) and two GaAsP detectors (DM 560 LP EM 524/42 (503-545) and DM 652 EM 600/45 (578-623)). Acquisition was controlled with NIS Elements software and data were analysed with Fiji and custom-written Python scripts.

Jurkat T cells expressing mCherry-fused TGFβ SNIPRs were seeded into microwells. Fluorescence labelling of recombinant TGFβ1 was performed by the following procedures. Recombinant TGFβ1 (10 µg) was dissolved in HEPES buffer (pH8, 100 mM) and then mixed with 5 equivalent of Alexa Fluor 647 NHS ester (Thermo Fisher Scientific A37573) (20 mg ml^−1^ in DMSO). After a one-hour reaction, excess dyes were removed by passing the solution through a spin desalting column (Zeba Micro Spin, Thermo Fisher Scientific). To induce SNIPR activation, Alexa Fluor 647-labelled TGFβ1 was added to the cells entrapped in microwells to a final concentration of 100 ng ml^−1^. Cells were stained with LysoTracker (Thermo Fisher Scientific L7526) for 30 min immediately before each imaging session at 6 h and 24 h. Live-cell imaging was performed using a 60× Plan-Apo oil objective (NA 1.42) on an Olympus Fluoview 3000 laser scanning confocal microscope. *Z*-stack images were acquired and data were analysed in Fiji.

### In vitro cell–cell communication assays

For in vitro detection of cell-produced TGFβ and VEGF, target cells were seeded in a flat-bottomed 96-well culture vessel at 20,000 per well in each cell type’s native medium. After 24 h of adherence and growth, the growth medium was aspirated and replaced with 40,000 T cells per well in human T cell medium. BFP reporter activation was determined by flow cytometry after 48 h of coculture. Similarly, the in vitro coculture in transwell cell system required 50,000 target cells per well in the transwell membrane (Corning 3388). After 24 h of incubation, T cells were seeded at 100,000 per well at the bottom of the well, and cells were incubated for 72 h before BFP reporter activation was determined by flow cytometry. For the production of TGFβ1 and VEGF by ELISA, target cells were seeded at 100,000 per well in a flat-bottomed 96-well culture vessel and incubated for 72 h. After incubation, a hTGFβ1 ELISA (Thermo Fisher Scientific BMS249-4) and hVEGF ELISA kit (Thermo Fisher Scientific KHG0111) were used following the manufacturer’s protocol. Following this same methodology, target cells were seeded and supernatant was collected for a Luminex TGFβ 3-plex Discovery Assay Multi Species Array (TGFβ1–TGFβ3) and a VEGF-A, VEGF-B and VEGF-C assay was performed by Eve Technologies. To generate orthoLigand sender cells, HeLa cells were transiently transfected by electroporation with a plasmid encoding an orthoLigand that was preceded by a modified serum albumin secretion tag. The cells were then grown for 48 h to allow for protein expression before collecting the medium. To eliminate any potential cell contamination, the medium was centrifuged at 500*g*. To assess the functionality of the orthoLigand, the conditioned medium from the sender cells was added to Jurkat cells, which served as the receiver cells. The cells were incubated for 24 h before being analysed for BFP expression. BFP signal was indicative of successful orthoLigand-mediated signalling between the sender and the receiver cells.

### Incucyte imaging

For in vitro live-cell imaging and killing assays, A375, A549 or M28 target cells bearing nuclear-localized mKate2 were seeded in flat-bottomed 96-well plates in their native medium. After 24 h, the medium was aspirated and immediately replaced with CD8^+^ T cells in RPMI 1640 (Gibco), 10% FBS, 50 U ml^−1^ penicillin–streptomycin and 30 U mll^−1^ IL-2 so as to maintain an expected 2:1 effector:target ratio at the start of the experiment. Plates were imaged using the Incucyte S3 Live-Cell Analysis System (Essen Bioscience) with three or four images collected per well and an imaging period of 4–6 h for at least 7 days of coculture. Cells were counted through automated segmentation of the fluorescent target cell nuclei using the Incucyte software.

### In vivo xenograft models

All animal work was performed under approval from the UCSF Institutional Animal Care and Use Committee (protocol AN177022-03C). All experiments used NOD.Cg- *Prkdc*^*scid*^*Il2rg*^*tm1Wjl*^/SzJ (NSG) (RRID:IMSR_JAX:005557) mice of the same sex, aged 8–12 weeks at the onset of the experiments. For all tumour models, dissociated cancer cells in 0.1 ml serum-free DMEM were implanted subcutaneously in the flank. T cells were administered retroorbitally in 0.1 ml PBS 7 days after tumour injection (5 days after sort). Experiments were performed with 1 × 10^6^ tumour cells and 6 × 10^6^ T cells per mouse, except for Fig. 5c, in which 1.5 × 10^6^ tumour cells and 9 × 10^6^ T cells were used. Tumour size was monitored by caliper, and for experiments using mouse–human cross-reactive CARs, mice were weighed to a precision of 0.1 g daily for one week after T cell injection and then at all subsequent tumour measurement time points. Mice were euthanized on tumour measurement along any axis of more than 20 mm, or on reaching a tumour volume of more than 2,000 mm^3^, where volume = ½ × largest axis × (smallest axis)^2^, or on weight loss of 15% below initial weight at tumour injection as specified in our IACUC-approved protocol, or when meeting humane euthanasia criteria for other reasons including impaired mobility, observable behavioural distress (laboured respiration, hunched appearance), or tumour ulceration covering more than 50% of the tumour surface area. Sample size was not pre-determined with statistical methods. Randomization and blinding were not performed.

### Statistics and reproducibility

Data points represent individual technical replicates unless otherwise stated. Technical replicates were performed as distinct samples. No statistical methods were used to predetermine sample size. For tumour curve comparison, area under curve measurements were compared; for mice that were euthanized before the end of the study, the final tumour volume measurement was prorated through the final time point. Comparison of mouse weight loss was performed on the day of peak weight loss for each individual experiment. The individual biological donors used are shown in Supplementary Table 1.

### Software and code availability

Chart plotting and statistical analysis were performed in GraphPad Prism 10. Flow cytometry and sorting were performed using BD FACSDiva, and post-hoc gating and analysis were conducted using FlowJo 10.8.0. Imaging colocalization analysis was performed with a custom-written Python script reporting the Pearson’s correlation coefficient (provided in Supplementary Data 1).

### Reporting summary

Further information on research design is available in the Nature Portfolio Reporting Summary linked to this article.

## Online content

Any methods, additional references, Nature Portfolio reporting summaries, source data, extended data, supplementary information, acknowledgements, peer review information; details of author contributions and competing interests; and statements of data and code availability are available at 10.1038/s41586-024-08366-0.

## Supplementary information

Supplementary Figure 1 Representative gating strategy for cell sorting and flow cytometry experiments.

Reporting Summary

Supplementary Table 1 Plasmid names and T cell donor information for each experiment described.

Supplementary Table 2 Description, part, and amino acid & DNA sequences for all plasmids described.

Supplementary Table 3 Description and sourcing of genetic components used.

Supplementary Table 4 List of antibodies used.

Supplementary Table 5 List of recombinant soluble factors for all experiments described.

Supplementary Table 6 List of pharmacological compounds used in receptor inhibition experiments.

Supplementary Data Supplementary File 1: Jupyter Notebook code for image colocalization analysis and example image.

## Source data

Source Data Fig. 1–5 and Source Data Extended Data Fig. 1–5

## Data Availability

Source data are provided with this paper.

## Code availability

The analysis code used for imaging colocalization analysis, along with an example image, is provided with this paper as Supplementary Data 1.
