# Supplementary material for: Engineered receptors for soluble cellular communication and disease sensing
Source: Nature. 2024 Nov 14;638(8051):805–13. doi: 10.1038/s41586-024-08366-0 (PMC11839477; doi:10.1038/s41586-024-08366-0)
Supplement: Supplementary file 1 — Representative gating strategy for cell sorting and flow cytometry experiments. [file 41586_2024_8366_MOESM1_ESM.pdf]

nature portfolio

https://doi.org/10.1038/s41586-024-08366-0

Supplementary information

Engineered receptors for soluble cellular communication and disease sensing

In the format provided by the
authors and unedited

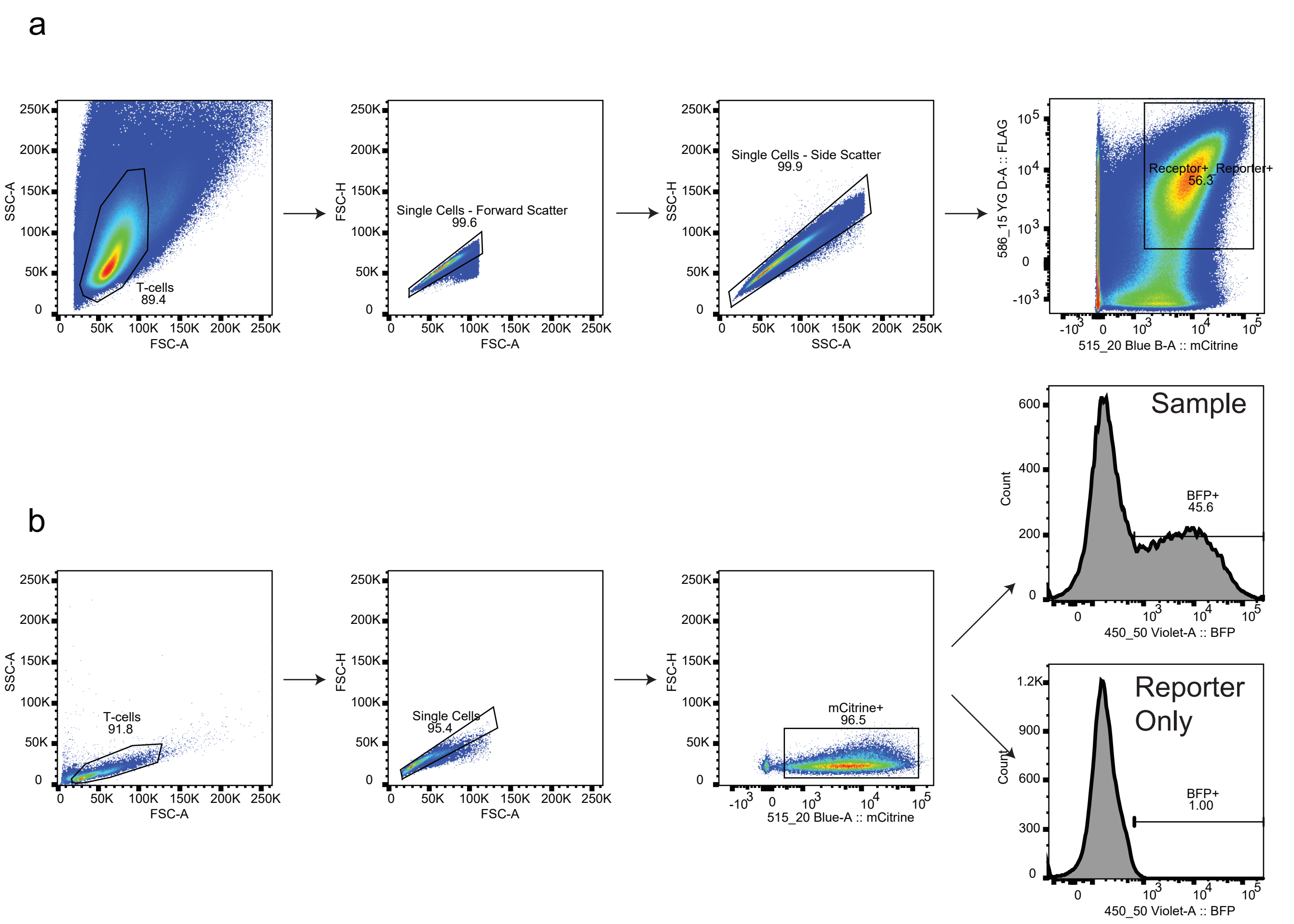
a
T-cells
89.4
SSC-A
FSC-A
Single Cells - Forward Scatter
99.6
FSC-H
Single Cells - Side Scatter
99.9
SSC-H
SSC-A
Receptor+ Reporter+
56.3
586_15 YG D-A :: FLAG
515_20 Blue B-A :: mCitrine
b
T-cells
91.8
Single Cells
95.4
mCitrine+
96.5
515_20 Blue-A :: mCitrine
Sample
BFP+
45.6
Count
450_50 Violet-A :: BFP
Reporter Only
BFP+
1.00

Figure S1: a) Representative gating strategy for T cell sorting. Dual transduced T cells harboring an epitope-tagged SNIPR and a SNIPR-inducible payload accompanied by a constitutive fluorescent protein (mCitrine) were gated by scatter profile followed by single cell separation on both forward and side scatter channels, and isolation based on dual expression of the epitope tag (V5 or FLAG) and the constitutive fluorophore on the reporter vector. **b)** Representative gating strategy for T cell activation analysis. T cells were gated on scatter profile and singlet status. Transduced T cells were selected based on expression of the constitutive fluorophore in the reporter vector, and activation was compared to a SNIPR-negative, reporter-only control.
