# Supplementary material for: Engineered receptors for soluble cellular communication and disease sensing
Source: Nature. 2024 Nov 14;638(8051):805–13. doi: 10.1038/s41586-024-08366-0 (PMC11839477; doi:10.1038/s41586-024-08366-0)
Supplement: Supplementary file 2 — Reporting Summary [file 41586_2024_8366_MOESM2_ESM.pdf]

nature portfolio

Corresponding author(s): Kole T. Roybal, David Baker.

Last updated by author(s): Oct 15, 2024

Reporting Summary

Nature Portfolio wishes to improve the reproducibility of the work that we publish. This form provides structure for consistency and transparency in reporting. For further information on Nature Portfolio policies, see our Editorial Policies and the Editorial Policy Checklist.

Statistics

For all statistical analyses, confirm that the following items are present in the figure legend, table legend, main text, or Methods section.

n/a	Confirmed	
☐	☒	The exact sample size (*n*) for each experimental group/condition, given as a discrete number and unit of measurement
☐	☒	A statement on whether measurements were taken from distinct samples or whether the same sample was measured repeatedly
☐	☒	The statistical test(s) used AND whether they are one- or two-sided<br>*Only common tests should be described solely by name; describe more complex techniques in the Methods section.*
☒	☐	A description of all covariates tested
☐	☒	A description of any assumptions or corrections, such as tests of normality and adjustment for multiple comparisons
☐	☒	A full description of the statistical parameters including central tendency (e.g. means) or other basic estimates (e.g. regression coefficient) AND variation (e.g. standard deviation) or associated estimates of uncertainty (e.g. confidence intervals)
☐	☒	For null hypothesis testing, the test statistic (e.g. *F*, *t*, *r*) with confidence intervals, effect sizes, degrees of freedom and *P* value noted<br>*Give P values as exact values whenever suitable.*
☒	☐	For Bayesian analysis, information on the choice of priors and Markov chain Monte Carlo settings
☒	☐	For hierarchical and complex designs, identification of the appropriate level for tests and full reporting of outcomes
☐	☒	Estimates of effect sizes (e.g. Cohen's *d*, Pearson's *r*), indicating how they were calculated

Our web collection on statistics for biologists contains articles on many of the points above.

Software and code

Policy information about availability of computer code

Data collection	Data was collected using FACSDiva version 9.0 CST version 3.5 and Incucyte S3 Software v2018B
Data analysis	Data was analyzed in FlowJo 10.8 and GraphPad Prism 9.

For manuscripts utilizing custom algorithms or software that are central to the research but not yet described in published literature, software must be made available to editors and reviewers. We strongly encourage code deposition in a community repository (e.g. GitHub). See the Nature Portfolio guidelines for submitting code & software for further information.

Data

Policy information about availability of data

All manuscripts must include a data availability statement. This statement should provide the following information, where applicable:

- Accession codes, unique identifiers, or web links for publicly available datasets
- A description of any restrictions on data availability
- For clinical datasets or third party data, please ensure that the statement adheres to our policy

Source data are provided with this manuscript.

Research involving human participants, their data, or biological material

Policy information about studies with human participants or human data. See also policy information about sex, gender (identity/presentation), and sexual orientation and race, ethnicity and racism.

Reporting on sex and gender	*Use the terms sex (biological attribute) and gender (shaped by social and cultural circumstances) carefully in order to avoid confusing both terms. Indicate if findings apply to only one sex or gender; describe whether sex and gender were considered in study design; whether sex and/or gender was determined based on self-reporting or assigned and methods used. Provide in the source data disaggregated sex and gender data, where this information has been collected, and if consent has been obtained for sharing of individual-level data; provide overall numbers in this Reporting Summary. Please state if this information has not been collected. Report sex- and gender-based analyses where performed, justify reasons for lack of sex- and gender-based analysis.*
Reporting on race, ethnicity, or other socially relevant groupings	*Please specify the socially constructed or socially relevant categorization variable(s) used in your manuscript and explain why they were used. Please note that such variables should not be used as proxies for other socially constructed/relevant variables (for example, race or ethnicity should not be used as a proxy for socioeconomic status). Provide clear definitions of the relevant terms used, how they were provided (by the participants/respondents, the researchers, or third parties), and the method(s) used to classify people into the different categories (e.g. self-report, census or administrative data, social media data, etc.) Please provide details about how you controlled for confounding variables in your analyses.*
Population characteristics	*Describe the covariate-relevant population characteristics of the human research participants (e.g. age, genotypic information, past and current diagnosis and treatment categories). If you filled out the behavioural & social sciences study design questions and have nothing to add here, write "See above."*
Recruitment	*Describe how participants were recruited. Outline any potential self-selection bias or other biases that may be present and how these are likely to impact results.*
Ethics oversight	*Identify the organization(s) that approved the study protocol.*

Note that full information on the approval of the study protocol must also be provided in the manuscript.

Field-specific reporting

Please select the one below that is the best fit for your research. If you are not sure, read the appropriate sections before making your selection.

☒ Life sciences ☐ Behavioural & social sciences ☐ Ecological, evolutionary & environmental sciences

For a reference copy of the document with all sections, see nature.com/documents/nr-reporting-summary-flat.pdf

Life sciences study design

All studies must disclose on these points even when the disclosure is negative.

Sample size	Sample size was selected based on prior experience with receptor / circuit systems to adequately reveal variation in the samples.
Data exclusions	For in vivo tumor measurement experiments, data presentation is cut off at the time point where mice begin to exhibit signs of advanced GVHD including severe alopecia or inflammation at the T cell injection site, even if additional data were collected on the mice not yet displaying these symptoms to inform further experiments.
Replication	All figures display technical replicates. Supplementary Figure S1d depicts biological replicates across donors for the key receptor designs. All key receptor designs were tested in multiple manufacturing batches (biological replicates) with multiple T-cell donors as described in Supplementary Table 1. Allowing for minor variation in T-cell fitness and transcriptional activity between donors, the relative effects of design modifications were replicated between batches.
Randomization	Mice were injected with T-cells in the order received from the breeding core, at the same experimental time point. Because the number of mice per cage typically did not match the experimental number of replicates, most experimental groups contained mice across multiple cages combined at random.
Blinding	Blinding was not relevant to these studies. Effect sizes greatly exceeded potential tumor measurement error via caliper.

Reporting for specific materials, systems and methods

We require information from authors about some types of materials, experimental systems and methods used in many studies. Here, indicate whether each material, system or method listed is relevant to your study. If you are not sure if a list item applies to your research, read the appropriate section before selecting a response.

Materials & experimental systems

n/a	Involved in the study
☐	☒ Antibodies
☐	☒ Eukaryotic cell lines
☒	☐ Palaeontology and archaeology
☐	☒ Animals and other organisms
☒	☐ Clinical data
☒	☐ Dual use research of concern
☒	☐ Plants

Methods

n/a	Involved in the study
☒	☐ ChIP-seq
☐	☒ Flow cytometry
☒	☐ MRI-based neuroimaging

Antibodies

Antibodies used	Myc-AF647. Cell Signaling. Clone 9B11. #2233S<br>Flag-PE. BioLegend. Clone L5. #637310<br>V5-PE. eBioScience. Clone 637310. #12-6796-42<br>CD19-BV421. eBioScience. Clone HIB19. #11-0199-41
Validation	All antibodies were provided by the manufacturer alongside Certificates of Analysis. All experiments involving antibody staining were performed with a target-negative control that was used to establish gating.

Eukaryotic cell lines

Policy information about cell lines and Sex and Gender in Research

Cell line source(s)	Lenti-X 293T: Purchased from Takara Bio. 632180. Female.<br>K562: Purchased from ATTC. CCL-243. Female.<br>Jurkat: Purchased from ATTC. TIB-152. Male.<br>A375: Obtained from A. Marson's laboratory at UCSF. Originally ATCC CRL-1619. Female.<br>A549: Obtained from UCSF Cell and Genome Engineering Core. CCLZR013. Male.<br>M28: originally obtained from B. Gerwin's laboratory at the National Cancer Institute. Gender not reported.<br>Caco-2: Purchased from ATTC. HTB-37. Male.
Authentication	Cells obtained from ATCC or the UCSF Cell and Genome Engineering Core were authenticated by the supplier.
Mycoplasma contamination	All cell lines tested negative for mycoplasma contamination (ATCC 30-1012K).
Commonly misidentified lines<br>(See ICLAC register)	No commonly misidentified cell lines were used in this manuscript.

Animals and other research organisms

Policy information about studies involving animals; ARRIVE guidelines recommended for reporting animal research, and Sex and Gender in Research

Laboratory animals	NSG Mice (NOD.Cg-PrkdcSCID Il2rgtm1Wjl/SzJ), 8-12 weeks of age at tumor injection.
Wild animals	Study did not involve wild animals.
Reporting on sex	Each experiment was performed in all-male or all-female mice to prevent sex-based confounds. Experimental batches were assigned to all-male or all-female mice based on animal availability.
Field-collected samples	The study did not involve samples collected from the field.
Ethics oversight	Animal works was conducted in accordance with the UCSF Institutional Animal Care and Use Committee (Protocol AN177022-03A).

Note that full information on the approval of the study protocol must also be provided in the manuscript.

Flow Cytometry

Plots

Confirm that:

☒ The axis labels state the marker and fluorochrome used (e.g. CD4-FITC).

☒ The axis scales are clearly visible. Include numbers along axes only for bottom left plot of group (a 'group' is an analysis of identical markers).

☒ All plots are contour plots with outliers or pseudocolor plots.

☒ A numerical value for number of cells or percentage (with statistics) is provided.

Methodology

Sample preparation	Flow cytometry was performed on cells in culture in 96well vessels. Staining procedures are described in methods. Briefly, cells were pelleted, resuspended in staining solution for 20-30 minutes at room temperature, washed, and resuspended in buffer for analysis.
Instrument	Sorters: BD Aria2 or Aria Fusion.<br>Cytometers: Mainly BD Fortessa X-50. Some BD Fortessa or Fortessa Dual.
Software	Flow data was analyzed in FlowJo 10.8 and exported in tabular form for plotting in Prism 9.
Cell population abundance	All cells were sorted in advance prior to experimentation. Most cells contained a constitutive fluorescent marker (typically mCitrine) which was detectable without necessitating staining. Data presented are gated on the marker-positive population (typically >97% of single cells in the parent gate).
Gating strategy	Cells were first gated on FSC/SCC for debris separation and FSC-A/FSC-H for single cell discrimination. Subsequently, cells were gated as follows:<br><br>CAR-only cells: Gated on CAR+ expression as indicated by Flag staining.<br>SNIPR + (CAR or reporter) cells: Gated on the constitutive marker on the reporter vector (either mCitrine for BFP reporter circuits, or BFP for CAR-expressing circuits) as well as Myc, Flag, or V5-stained receptor.<br><br>Gates were determined based on identically stained untransduced T-cells. For BFP reporter experiments, the BFP+ gate during flow cytometric analysis was set based on the BFP channel signal of reporter-only or untransduced T-cells that underwent the identical staining and preparation procedure.

☒ Tick this box to confirm that a figure exemplifying the gating strategy is provided in the Supplementary Information.
