# Supplementary material for: Engineered receptors for soluble cellular communication and disease sensing
Source: Nature. 2024 Nov 14;638(8051):805–13. doi: 10.1038/s41586-024-08366-0 (PMC11839477; doi:10.1038/s41586-024-08366-0)
Supplement: Supplementary file 9 — Supplementary File 1: Jupyter Notebook code for image colocalization analysis and example image. [file 41586_2024_8366_MOESM9_ESM.zip › Supplementary File 1/ReadMe.pdf]

1. **System Requirements**
    a. **Software dependencies and operating systems**
        i. This script is compatible with any operating system that can run JupyterLab
        ii. Package dependencies:
            1. Pandas
            2. Numpy
            3. Nd2reader
            4. opencv-python
            5. scikit-image
            6. scipy
            7. scikit-learn
    b. **Versions the software has been tested on**
        i. OS: Windows 10 Pro
        ii. JupyterLab 4.2.5 (via Anaconda 2.6.2)
        iii. Package dependencies:
            1. Pandas 2.0.3
            2. Numpy 1.24.4
            3. Nd2reader 3.3.0
            4. opencv-python 4.7.0.72
            5. scikit-image 0.19.1
            6. scipy 1.8.0
            7. scikit-learn 1.0.2
    c. **Non-standard hardware requirements: None**
2. **Installation guide**
    a. **Instructions**
        i. Launch JupyterLab via your preferred method
        ii. In JupyterLab, open Pearson Analysis.ipynb
    b. **Typical install time: < 1 minute**
3. **Demo**
    a. **Instructions to run on data**
        i. In code block 1, replace the definition of the **directory** variable with the path to the directory containing one or more images.
            1. Image specifications:
                a. .nd2 format
                b. 3 channels corresponding to 488 nm, 561 nm, and 640 nm emission
        ii. Run code block 1
        iii. Run code block 2
        iv. Run code block 3
            1. Wait for the "Finished analyzing images" message to appear in the output.
        v. In code block 4, replace the definition of the **output_file** variable with the path and file name of the summary .csv file to which you would like to write the correlation metrics for each image.

vi. Run code block 4

b. **Expected output**

	A	B	C	D	E	F	G	H
1		Filename	Timepoint	Well	Number	Correlation: 488_56	Correlation: 488_64	Correlation: 561_64
2	0	/home/thomschl/Pap	15min	3	1	0.7742290637	0.1379996009	0.2454052289

c. **Expected run time for demo: < 1 minute**

4. **Instructions for use**

   a. **How to run the software on your data**

      i. Ensure that your image files comply with the specifications listed above in 3.a.i.1.

      ii. Place your .nd2 image files into a single directory.

      iii. Follow the instructions in section 3 above, replacing the default **directory** path with the path to your data.

      iv. Run the code.
